# Migraine: Experimental Models and Novel Therapeutic Approaches

**DOI:** 10.3390/ijms20122932

**Published:** 2019-06-15

**Authors:** Giuseppe Tardiolo, Placido Bramanti, Emanuela Mazzon

**Affiliations:** IRCCS Centro Neurolesi “Bonino Pulejo”, 98124 Messina, Italy; giuseppe.tardiolo@irccsme.it (G.T.); placido.bramanti@irccsme.it (P.B.)

**Keywords:** migraine, animal models, experimental approaches

## Abstract

Migraine is a disorder affecting an increasing number of subjects. Currently, this disorder is not entirely understood, and limited therapeutic solutions are available. Migraine manifests as a debilitating headache associated with an altered sensory perception that may compromise the quality of life. Animal models have been developed using chemical, physical or genetic modifications, to evoke migraine-like hallmarks for the identification of novel molecules for the treatment of migraine. In this context, experimental models based on the use of chemicals as nitroglycerin or inflammatory soup were extensively used to mimic the acute state and the chronicity of the disorder. This manuscript is aimed to provide an overview of murine models used to investigate migraine pathophysiology. Pharmacological targets as 5-HT and calcitonin gene-related peptide (CGRP) receptors were evaluated for their relevance in the development of migraine therapeutics. Drug delivery systems using nanoparticles may be helpful for the enhancement of the brain targeting and bioavailability of anti-migraine drugs as triptans. In conclusion, the progresses in migraine management have been reached with the development of emerging agonists of 5-HT receptors and novel antagonists of CGRP receptors. The nanoformulations may represent a future perspective in which already known anti-migraine drugs showed to better exert their therapeutic effects.

## 1. Introduction

Migraine is a complex neurological disease considered the primary headache disorder leading to disabling conditions [1]. The last International Classification of Headache Disorders (3^rd^ edition) describes migraine as a recurrent headache disorder manifesting as a unilateral and throbbing headache with pain intensity from moderate to severe [2]. Common symptoms observed are photophobia and phonophobia, nausea and/or vomiting [2]. Migraine can be distinguished in episodic migraine (EM) and chronic migraine (CM). EM is defined whether the headache days per month are less than 15, and CM, whether headache days are equal or more than 15 for a period of more than three months. Moreover, migraine appears to occur more in women than in men [3]. In addition, it is estimated that about 30% of patients experience an aura that consists in a short period of visual, sensory, or motor disturbances [4]. Mechanisms involved in migraine are not yet entirely clarified. To date, it is thought that the genesis of pain occurs by activation of the trigeminovascular system (TGVS). This system is composed of the cranial vasculature, the trigeminal nerve and the trigeminal nucleus caudalis (TNC). TGVS plays an important role as a major control center in regulating the cerebral blood flow and it is believed as a key conduit for pain transmission [5]. The activation of trigeminal sensory nerve endings induces the release of vasoactive agents, such as calcitonin gene-related peptide (CGRP), substance P and neurokinin A, resulting in vasodilation and dural plasma extravasation, leading to neurogenic inflammation [5]. Current migraine treatments regard the use of drugs aimed to decrease the frequency, severity, and last of migraine attacks [6]. In case of mild attacks, medications such as acetaminophen and aspirin are used, whereas triptans or dihydroergotamine (DHE) are used for the treatment of a moderate to severe migraine [7,8]. The current pharmacological treatment used in migraine is summarized in Table 1. Our current knowledge about the pathophysiology of this complex disorder is based mostly on animal models developed to study the nociceptive pathways of the TGVS and their ascending projections to the brainstem and the diencephalic nuclei. These models are mostly based on modifications operated in order to try mimicking headache symptoms, requiring manipulations to activate the trigeminal nerve or dural nociceptors [5,9,10]. Although animal models show shortcomings per se, due to difficulties in reflecting all hallmarks of migraine, they have been used as a screening tool for the development of novel anti-migraine drugs, in which serotonin (5-HT) and CGRP receptors have importantly contributed as targets, showing to be involved in pathways that result in headache attacks [11]. Furthermore, drug delivery systems based on the use of formulations composed of nanoparticles could be considered a new attempt to improve the effects of drugs used in migraine treatments. The present manuscript has been generated using PubMed as source of information, with the aim to offer an overview of murine models developed to study migraine pathophysiology. For the promising results obtained in migraine treatment, in this manuscript the pharmacological targets 5-HT and CGRP receptors were evaluated. In addition, the database ClinicalTrials.gov [12] has been used as source of information for the new emerging treatments using agonists of 5-HT receptors and new antagonists of CGRP receptors. Validated animal models of migraine are summarized to provide an overview. At last, new therapeutic strategies and nanoparticles tested in experimental models performed on rats or mice in the last years were considered.

## 2. Pharmacological Targets in Migraine Treatment: 5-HT and CGRP Receptors

Research advances are increasingly focusing on the development of anti-migraine drugs. In this context, 5-HT and CGRP receptors have been mainly investigated. 5-HT receptor agonists and CGRP receptor antagonists are providing an important contribution in emerging treatments aimed to counteract migraine attacks [15]. The new emerging treatments, approved or still looking for approval by FDA, are summarized in Table 2.

Serotonin or 5-hydroxytryptamine (5-HT) is a monoamine neurotransmitter, widely distributed both centrally and peripherally, in the human body. It is primarily found in the enteric nervous system located in the gastrointestinal tract. It is also produced in the central nervous system (CNS), specifically in the raphe nuclei where neurons containing 5-HT have been observed [16]. 5-HT biological functions are multiple and complex and its eventual response mainly depends on the nature of the 5-HT receptors implicated [17]. Seven types of 5-HT receptors have been identified, respectively named: 5-HT_1_, 5-HT_2_, 5-HT_3_, 5-HT_4_, 5-HT_5_, 5-HT_6_ and 5-HT_7_. 5-HT_1_ and 5-HT_2_ receptors are respectively divided into the following subtypes: 5-HT_1A_, 5-HT_1B_, 5-HT_1D_, 5-HT_1E_, 5-HT_1F_ and 5-HT_2A_, 5-HT_2B_, and 5-HT_2C_ [18,19]. Receptors functional characteristics depend on their molecular structure, being G-protein-coupled receptors such as: 5-HT_1_, 5-HT_2_, 5-HT_4_, 5-HT_5A_, 5-HT_6_ and 5-HT_7,_ or integral to an ion channel such as 5-HT_3_ [20]. All 5-HT_1_ receptor subtypes are coupled to Gi/o, a protein that predominantly inhibits adenylyl cyclase activity, with consequent inhibition of the release of neurotransmitters and reduction in neuronal firing [17,20]. Triptans are agonists of 5-HT_1B/1D_ receptors and showed to be efficacious in the acute treatment of migraine, thus providing an indirect proof suggesting the involvement of serotonin in the pathogenesis of migraine [21,22]. An increased production of 5-HT seems to occur in migraineurs brain compared to control subjects, that might lead to cortical hyperexcitability [23]. A reduction in blood concentrations of 5-HT was observed in subjects with migraine in the absence of aura during the headache phase [24]. Another study indicates that low 5-HT_4_ receptor binding, that suggests a high 5-HT concentration in the brain, can be considered a trait marker of migraineurs rather than a risk factor that can lead to a conversion from EM to CM. Higher concentrations of 5-HT could lead to an enhancement of the susceptibility to migraine attacks. Therefore, a reduction of 5-HT concentrations could reveal effective to treat migraine, indicating that further studies involving other 5-HT receptor subtypes and modulation of cerebral 5-HT concentrations in migraine subjects are required [25]. The increased understanding of migraine pathophysiology recently resulted in developing novel molecules that are under investigation as emerging options to be used in therapeutics. The novel ditans are serotonin 5-HT_1F_ receptor agonists, a class of drugs that differentiate from the known triptans in showing high selectivity at receptors. The ditan Lasmiditan is currently under investigation in migraine acute treatment [15].

CGRP is a multifunctional neuropeptide that appears to play an important part in migraine mechanism and its origin was predicted observing the alternative splicing of the calcitonin gene [26]. This neuropeptide is known as one of the most potent vasodilators [27]. Two isoforms have been characterized, α-CGRP and β-CGRP. The isoform α is principally expressed in primary sensory neurons, whereas the isoform β is mainly found in intrinsic enteric neurons [27]. The mature form of this neuropeptide is composed of 37 amino acids, and its expression has been particularly noticed in sensory neurons of the dorsal root ganglia (DRG) and trigeminal ganglion (TG) [26,27]. The mature form is stored in vesicles localized in the terminal region of central and peripheral nerve endings. There, their content may be secreted in the dorsal spinal cord or in various peripheral tissues, especially surrounding blood vessels which may modulate vascular tone [28]. In addition, the presence of nociceptors network positive to CGRP in rodent and human meningeal vessels has been observed [29,30], and about 40–50% of TG neurons are positive to CGRP [31,32]. Moreover, in areas of the CNS, such as hypothalamus, thalamus, periaqueductal gray, superior and inferior colliculi, amygdala, trigeminocervical complex (TCC) and the cerebellum, the expression of CGRP has been observed [33,34]. These mentioned brain areas may be associated with migraine pathophysiology, considering the capability of CGRP to change synaptic and neuronal activity at the TCC, and transmission of nociceptive signals to the thalamus and cortical areas [35,36]. The structure of CGRP receptor is a complex of proteins composed as follows: a G-protein-coupled receptor named the calcitonin receptor-like receptor (CLR) [37]; a single transmembrane accessory protein named receptor activity-modifying protein 1 (RAMP1) [38] (needed to establish the binding of CGRP to CLR), and the receptor component protein (RCP) [39] that characterizes the G-protein associated with the receptor. The expression of the subunits that compose the CGRP receptor complex has been observed in peripheral and central sites [32], e.g., in cell bodies in TG, in the periaqueductal grey, and in the TNC [40,41]. Nevertheless, it is not yet completely established whether the assembly of all these subunits composing the fully functional receptor form is performed in these anatomical structures. Despite this, the expression of the functional form of CGRP-receptor complex has been observed in vascular smooth muscle cells in arteries and arterioles (also in those of the cranial circulation), as suggested, both in vitro and in vivo [27], by the powerful vasodilatory effect of CGRP in these blood vessels. To date, CGRP is considered a new important pharmacological target for migraine treatment [15]. CGRP antagonists act by inhibiting vasodilation and neurogenic inflammation through release blockage of CGRP in the migraine pathway [42].

Emerging CGRP receptor antagonists, such as ubrogepant and rimegepant, are currently under assessment in therapeutics [15]. In addition, the development of new CGRP receptor or ligand antagonists is ongoing, in which monoclonal antibodies (mAbs) such as fremanezumab, galcanezumab and erenumab (all approved by FDA in 2018 for EM or CM), are opening a new approach in therapeutic strategy, representing a valuable support to the solutions already available [15].

## 3. Animal Models of Migraine

Animal models have been developed with the aim to comprehend migraine disorder, even though all of them show shortcomings. To be considered reliable, animal models should display a similar etiology and phenotype to human migraine. Although this disorder is considered complex with a variable phenotype, there is currently no animal model able to replicate all its features [52]. In this paragraph, we consider murine models developed to study the migraine disorder. In Table 3, we summarized the current animal models of migraine in which rats or mice were used.

Currently, animal models focus on activation of nerves in TCC. Nociceptors are located in the terminal structures of meningeal and trigeminovascular afferents deriving from the ophthalmic division of the trigeminal nerve that innervate intracranial structures sensitive to pain, such as the dura mater and meningeal vasculature, large cerebral arteries and the paranasal sinuses. Headaches similar to migraine can be caused by stimulation of nerves that innervate these structures [53,54]. Animal models based on chemical provocations that use different vasodilating agents are probably the most investigated in preclinical research. The administration of a mix of inflammatory mediators, named “inflammatory soup”, e.g., using a mixture composed by prostaglandin (PGE2), histamine, 5-HT and bradykinin, has been used to stimulate meningeal and trigeminovascular nociceptors [55]. In this model, the inflammatory soup can be administered by injection using a micro-catheter placed into the cisterna magna through the atlanto occipital membrane of animals that received anesthetics. This injection of chemicals induces the activation of the primary sensory fibers supplying the meninges. In addition, topical application on the dura mater of rats is also used causing a reversible cephalic mechanical sensitivity [56,57,58]. Potential limitations of this technique are linked to the use of chemicals that can compromise the functionality of the blood-brain-barrier (BBB), resulting in activation of central sites directly rather than synaptically by the activation of meningeal afferent fibers [52].

Another model widely used, based on nitroglycerin (NTG), nitric oxide donor glycerol trinitrate, intravenously or intraperitoneally administered, has been studied as method to provoke migraine-like pain [52]. In rodents, hyperalgesia provoked by NTG has been used to develop a model to study sensory hypersensitivity associated with migraine [59,60]. The infusion of NTG in mice caused thermal and mechanical allodynia, symptom reversed by sumatriptan [59]. Moreover, in a transgenic mouse model of familial migraine that was studied, animals that expressed a human migraine gene, casein kinase 1δ, in which a major sensitivity to hyperalgesia induced by NTG, compared to controls, was observed [61]. In addition, NTG-administered in mice was able to induce aversion to light and increased meningeal blood flow [60,62]. This mouse model was used to develop a test able to model the progression of the disorder from an acute to a chronic condition by means intermittent injection of NTG. This modality of treatment evoked acute hyperalgesia and developed a progressive basal hypersensitivity to mechanical stimulation [63]. Both acute hyperalgesia and hypersensitivity were blocked by topiramate and propranolol, whereas hyperalgesia was inhibited by sumatriptan, suggesting that this model may be considered in the screening of novel therapies in migraine treatment [64]. The use of NTG in rodents may effectively model migraine-like symptoms [65], even though doses, type of administrations and the determination of the time of observation need to be carefully monitored when animal models based on NTG are adopted for the study of the TGVS [52].

Medication overuse headache (MOH) is a condition that in predisposed subjects affected by migraine or tension-type headache typically occurs. In these conditions, an overusing of drugs such as triptans and opioids, e.g., for more than 10 days per month over 3 months, may transform the headache from an episodic to a chronic state. In rodents, MOH may be used as a model in which repeated administration of these drugs induced a long-lasting state of latent sensitization [66]. It has been reported that TEV-48125, a humanized CGRP antibody, inhibited cutaneous allodynia both induced by bright light stress and NO donor in a MOH model, in which rodents were prior treated with sumatriptan or morphine, thus highlighting the importance of this model in discovering migraine medications [67].

In recent decades, several gene mutations have been correlated with some forms of severe and rare migraine by the use of an approach based on classic linkage analysis. This analysis contributed to sustain the hypothesis that an inherited trait is implicated in this disorder. A translational step offered by transgenic mouse technologies, in supporting the understanding about the pathophysiology of migraine and the evaluation of new therapeutic targets, is represented by behavioral characterization of genetic models of migraine [68]. The familial hemiplegic migraine (FHM) is a rare monogenic migraine, in which affected subjects show a severe hemiplegic aura accompanied by weakness perceived on one side of the body. Specifically, three gene mutations were identified to date, CACNA1A (FHM1), ATP1A2 (FHM2), and SCNA1A (FHM3), encoding for subunits of ion channels and transporters that show to have a role in neurotransmission [69]. These mutations, respectively encode for subunits of voltage-gated calcium channels, sodium-potassium ATPases, and voltage-gated sodium channels [70]. In this frame, genetic models of migraine have been created by inserting human mutated genes in the mouse genome obtaining knock-in (KI) mouse models, respectively, two of FHM1 [71,72], and one of FHM2 [73]. The humanized FHM1 KI mouse models contain gain of function missense mutations (R192Q or S218L) in the CACNA1A gene, one of the most investigated genes whose product is the pore-forming α1A subunit of Cav2.1 channels (P/Q type) (55). S218L mice showed a phenotype similar to a severe clinical phenotype of patients showing the same mutation (FHM1 S218L) [72]. In R192Q mice, decreased CGRP-immunoreactivity was observed when compared to controls, in cells of TG and in the superficial laminae of the TCC, suggesting that alteration in the expression of CGRP is induced by FHM-1 CACNA1A mutation [74]. These models showed decreased neuronal response to nociceptive activation of the TGVS in comparison with controls [75], suggesting that they show different reactions to common nociceptive signals observed in migraine. In addition, enhanced susceptibility in showing spontaneous pain behaviors correlated to nociceptive headache and photophobia was highlighted [76,77]. Only one KI mouse model for FHM2 expressed the loss of function W887R missense mutation in ATP1A2 [73], whereas no FHM3 KI mouse model has been developed yet.

In a study based on a rodent model, during the process of evaluating baseline periorbital von Frey thresholds, a male rat affected by spontaneous episodic trigeminal allodynia was discovered [78]. This characteristic was noticed by episodic alteration of periorbital pain threshold. The mating demonstrated that this trait is inheritable in both sexes and with a diversity of phenotypes. Some animals showed a similarity to chronic migraineurs, with thresholds lower than normal for more than two weeks per month. Other animals showed a similarity to episodic migraine, manifesting periods of normal thresholds and periods of lower thresholds. Chemicals such as sumatriptan, ketorolac and DHE were tested in order to validate this model, temporarily reversing the pain thresholds. Furthermore, the treatment with valproic acid for a period of one month blocked spontaneous changes in trigeminal allodynia. After the discontinuation of the treatments, the animals returned to the initial baseline. This study might be considered a unique model of spontaneous allodynia with phenotypes similar to migraine, providing a possible predictive model for drug development and for the investigation of the pathophysiology of spontaneous episodic trigeminal pain disorders [78]. Research based on these genetic models offers an advantage due to the fact that animals are manipulated in order to have predefined phenotypes similar as possible to migraine features. Nonetheless, the disadvantages might regard the common polygenic forms of this disorder in the general population, in which these specific mutations might not be relevant.

Migraine aura and cortical spreading depression (CSD) are transient neurological deficits highlighted in around 30% of migraineurs [79]. CSD is a phenomenon described as a slow wave of depolarization of neuronal and glial cells in the cortex that can be induced experimentally. In rodent models, injections of KCl showed to initiate this phenomenon in the cortex. CSD visualization is operated measuring the electrical activity of cortical neurons by means implanted microelectrodes or changes in cerebral blood flow through laser doppler flowmetry [52]. Furthermore, CSD can be also induced by electrical or mechanical stimulation of the cortex [80]. Progresses in the study of CSD mechanisms have focused to elucidate whether it plays a part in provoking the trigeminovascular activation, resulting in migraine triggering. In rodents, studies based on imaging showed that CSD induces vasodilation of meningeal blood vessels [81], and enhanced neuronal activation at the level of trigemino nuclear complex and in higher cerebral areas of the trigeminal pain pathway [82]. Considering that migraine frequently occurs without aura, the mechanisms of CSD require further studies to be better clarified and for drug discovering in migraine aura treatment.

## 4. New Therapeutic Strategies in Experimental Models

The first therapeutic approach to migraine is generally symptomatic and aimed to alleviate acute pain, and medications are more effective whether quickly administered. Notwithstanding, there are advances in the development of anti-migraine drugs, and there is a growing need in researching novel therapeutic approaches aimed to treat more effectively migraine compared to actual treatments. In this context, we therefore consider studies on animal models developed with this perspective in this study. The most relevant findings of new compounds and nanoparticles in experimental models are summarized in Table 4.

In a recent study, Moye et al. [92] studied the efficacy of SNC80, a δ opioid receptor (DOR) agonist, in mouse models that replicated different headache disorders. In these models, mice were managed in order to induce CM, post-traumatic headache (PTH), MOH, and opioid-induced hyperalgesia (OIH) [92]. In CM model, mice received NTG by the intraperitoneally intermittent administration. In PTH, mice received isoflurane to be mildly anesthetized and then underwent the closed head weight-drop method in order to induce mild traumatic brain injury, and two weeks after PTH was modelled by low NTG dose intraperitoneally. To model MOH and OIH, animals received intraperitoneally treatment using respectively sumatriptan or morphine. In CM model, animals treated with NTG showed basal peripheral and cephalic hypersensitivity. To evaluate the effect of the activation of DOR, an acute treatment of SNC80 was performed 24 h after the last injection of NTG. This treatment showed a relevant attenuation of peripheral and cephalic allodynia compared to controls, indicating that pain associated with CM was blocked by DOR activation. In PTH model, basal peripheral and cephalic hypersensitivity were developed in mice treated with NTG compared to controls. Twenty-four hours after the last NTG injection, cephalic allodynia was inhibited by performing an acute SNC80 treatment, indicating that also in this case, the pain associated with PTH was attenuated by DOR activation. In MOH model, basal hind paw and cephalic hypersensitivity were developed in mice treated with chronic administration of sumatriptan. Twenty-four hours after the final injection of medication, mice received an acute treatment with SNC80 that resulted in allodynia attenuation, suggesting that MOH induced by overuse of sumatriptan can be inhibited by DOR activation. In OIH model, mice received chronic treatment with morphine, showing basal hind paw and cephalic hypersensitivity, an effect that was also observed 18–24 h after the last drug injection. After, SNC80 was administered resulting in allodynia effect attenuation induced by morphine treatment. Furthermore, it has been observed that chronic daily administration of SNC80 causes a limited form of MOH, less severe in comparison with mice treated with sumatriptan. These results suggest that DOR agonists might represent a novel therapeutic approach in the treatment in diverse headache disorders showing a different etiology [92].

Pradhan et al., have investigated the therapeutic potential of δ-opioid receptor agonists in mouse migraine models induced with acute and chronic doses of NTG [93]. Animals were treated with three different δ-opioid receptor agonists, SNC80, ARM390 or JNJ20788560, about 1 h 30 min following NTG injection. These receptor agonists were able to significantly reduce NTG-evoked hyperalgesia. In addition, a model of migraine aura was induced by continuous application of KCl in order to evaluate the effects of SNC80 on evoked CSD. SNC80 in the 1 h time interval following administration, was able to reduce the number of CSD events. These data showed the therapeutic ability of the δ-opioid receptor as a promising therapeutic target for migraine [93].

Hoelig et al., studied the effectiveness of NOX-L41, a CGRP-neutralizing mirror-image (L-) aptamer (termed Spiegelmer), in a rat model of electrically evoked meningeal plasma protein extravasation (PPE) [94]. In this study, the authors have tested a Spiegelmer, a molecule synthesized chemically consisting of mirror-image oligonucleotide, which is able to bind to a pharmacologically relevant target molecule. Animals received NOX-L41 as a single dose intravenously or subcutaneously, showing a plasma half-life of 8 h. Furthermore, by means pharmacodynamic studies, after a single administration an extravasation of NOX-L41 from blood vessels in the dura mater and inhibition of neurogenic meningeal PPE for at least 18 h was observed. The Spiegelmer action consisted in binding CGRP, a neuropeptide that promotes meningeal vasodilation, inhibiting CGRP in signaling at its receptor. NOX-L41 showed increased affinity and selectivity for both isoforms, α and β, of human CGRP than a previous studied NOX-C89. The capacity of NOX-L41 to extravasate in surrounding tissues of dural circulation, in order to interact with perivascular vasodilating agents, results in the need to develop compounds able to antagonize CGRP, preventing neurogenic inflammation. This study suggests further the involvement of CGRP in neurogenic PPE, indicating NOX-L41 as a future potential compound for the treatment or prevention of migraine [94].

In recent times, the development of drug delivery systems using nanoparticles has received an increased attention from the scientific community. In this context, Girotra et al. [95,96,97,98,99] conducted studies on nanoparticulates formulations with the aim to increase the brain targeting of pharmacological anti-migraine drugs. Here, we consider some of their investigations.

A dual therapeutic approach was performed on rats and mice in the development of brain-targeted rizatriptan benzoate-loaded solid lipid nanoparticles (RB-SLNs), with the purpose to improve the drug potentiality in counteracting migraine [95]. The formulations have been elaborated by fabricating optimized solutions to obtain particle sizes showing sufficiently high entrapment efficiency and drug release in about 8 h. To evaluate the typical symptoms related to migraine, acetic acid-induced writhing test and light/dark box model were respectively used to induce hyperalgesia and light aversive behavior. Animals that received optimized RB-SLNs by oral administration showed a decrease in migraine-related hallmarks. After 2 h of oral drug treatment, pharmacodynamic evaluations showed that in rats, the brain uptake potential of optimized RB-SLNs was about 18.43-folds greater compared to the free form of the pure drug, whereas in mice showed to cross the BBB resulting in an improvement of its anti-migraine effectiveness. These findings indicate that RB-SLNs showed an improvement in brain target ability, thus providing a potential approach for migraine management [95].

In another study, an innovative approach has been conceived by developing chitosan solid lipid nanoparticles (SLN) that contained sumatriptan succinate (SS) [96]. The SLN formulations optimized in brain targeting. The optimization of the formulations was accomplished by multi-level design factorial in order to obtain a minimize size particles with a high entrapment efficiency and drug concentrations. Rats received the formulations, previously dispersed in deionized water, by oral administration. Behavioral studies indicating a reduction in hyperalgesia in the acetic acid induced writhing test and reduced aversion to light in light/dark box model. The treatment with formulations showed a major availability of SS in the brain in comparison with controls. These results suggest that formulations orally administered consisting in hydrophilic drug SS, loaded in chitosan SLN, were able to cross the BBB, allowing the drug in exerting its pharmacological activity in the brain. Considering their data, nanoparticulate drug delivery systems might represent a future approach to cross the BBB and to improve brain targeting of medications in migraine therapeutics [96].

A further study has been conducted on another formulation for brain targeting of SS, with the aim to evaluate the optimal therapeutic effect of the drug in migraine. For this purpose, nanoparticulate drug delivery system using poly (butyl cyanoacrylate) (PBCA) and bovine serum albumin linked with apolipoprotein E3 (BSA-ApoE) was used [97]. SS was incorporated in the BSA-ApoE NPs and compared with the same drug loaded polysorbate 80 coated optimized PBCA NPs to determine the brain uptake potential of these formulations. The central composite design was used for the formulation of PBCA NPs optimized with minimum particle size, maximum entrapment efficiency along with the sustained drug release. Also, in this study, animals were treated and assessed as described in the previous investigations, and behavioral studies showed similar improvements. The treatments with the nanoformulations prepared in this study showed a high brain/plasma drug ratio 2 h after the oral drug administration. The data obtained by the authors suggest that BSA-ApoE NPs showed a better activity than polysorbate 80 coated PBCA NPs for brain targeting of SS. This technique might offer a perspective, as improved therapeutic approach for the treatment of migraine [97].

In another study concerning the use of nanoparticles, Poly (D,L Lactide-co-Glycolide) (PLGA)/poloxamer nanoparticles (NPs) of the hydrophilic medication zolmitriptan were developed [98]. Randomized factorial design to obtain the critical quality characteristics of minimized particle size and maximized encapsulation efficiency was applied. To determine the brain uptake potential, rats received optimized zolmitriptan encapsulated PLGA NPs as oral administration, and at the different time points, plasma and brain samples were collected. Acetic acid induced writhing test and light/dark box model were respectively used to induce hyperalgesia and aversion to light in mice. After the treatment, the in vivo studies for determining the brain uptake potential showed a 14.13-fold increase in the drug delivered to the brain from the NPs as compared to the free drug. Behavioral tests showed a decrease in the number of writhings, and a significant reduction of light aversion compared to controls. These data suggest that PLGA NPs containing zolmitriptan may represent a future tool in providing systems to cross the BBB for drug delivery that can exert an enhanced anti-migraine effect [98].

Another investigation has been performed using pharmacophore modeling [99]. This technique led to the identification of nystatin as compound active against the receptors iGluR5 kainate receptor (1VSO), CGRP (3N7R), β_2_ adrenoceptor (3NYA) and Dopamine D_3_ (3PBL). Following this result, brain targeted chitosan nanoparticles containing nystatin were prepared, later intraperitoneally administered in rats in order to evaluate brain targeting efficacy. Confocal laser scanning microscopy showed a higher nanoparticles accumulation in the brain than liver and spleen. After treatment with nystatin nanoformulations, behavioral tests performed in mice showed a reduction in hyperalgesia, photophobia and phonophobia compared to controls. This study represents the first approach of the therapeutic potential of nystatin nanoformulations, suggesting their future application in the treatment of migraine [99].

Wang et al., developed a novel ester derivative of gastrodin (Gas), termed Gas-D, and studied its effectiveness in a model of NTG induced migraine in rats [100]. Gas is a compound obtained by *Gastrodiae Rhizoma* (known in China, also as Tianma), and it has already been used for the treatment of migraine. Rats received Gas-D intragastrically, and subsequently treated by NTG subcutaneously 1 h after the final treatment. The pretreatment with Gas-D showed a reduction in head-scratching behavior, previously induced by NTG treatment. Results obtained from this study require further pharmacokinetic and pharmacodynamic investigations, suggesting that Gas-D, thanks to its anti-migraine effect, might be considered as a future candidate for migraine treatment [100].

Zhao et al., performed a comparative study evaluating two traditional Chinese drugs, gastrodin and ligustrazine, in a rat model of nociceptive durovascular trigeminal activation [101]. In this study, Chinese medications were compared to two Western approaches with propranolol and levetiracetam. Animals underwent surgical procedures for drug administration by femoral vein cannulation. An electrode was applied onto the dura mater above the middle meningeal artery and used to record the electrical stimuli in the TCC. When a reliable baseline to dural electrical stimulation was established, either gastrodin, levetiracetam, ligustrazine, or propranolol was administered. The treatment showed that gastrodin was able to inhibit nociceptive dural-evoked neuronal firing in the TCC, whereas ligustrazine showed no relevant effect on spontaneous activity in the TCC. To perform a comparison with the Chinese drugs, the established migraine preventive propranolol and the ineffective compound levetiracetam were used. As a result, propranolol showed a significant inhibition of dural-evoked responses, whereas the use of levetiracetam showed no effect. Their data suggest that gastrodin showed potential as an anti-migraine treatment, and on the contrary, ligustrazine appeared less promising. Therefore, these findings suggest further investigations about the use of gastrodin in migraine treatment. In addition, the results indicate the usefulness in exploring the traditional Chinese medicine approaches as signposts in developing new drugs for migraine [101].

In the last years, further experimental models have been developed using different approaches. Electrical stimulations have been used in experimental models with the purpose to better understand the mechanisms of migraine. Recently, Zhang et al. developed a repetitive electrical stimulation rat model [102]. In this study, the authors showed a dynamic model that upon stimuli of the dura mater, the TG begins to increase the production of the vasoactive neuropeptide pituitary adenylate cyclase-activating peptide (PACAP). PACAP is released from periphery terminals of the TG to innervating areas such as the dura mater, leading to vasodilation. PACAP is transported through central terminals to the TNC, where PACAP binds to PACAP-preferring type 1 (PAC1) receptor and triggers the excitation of nociceptive neurons with a consequent further increase in PACAP. The new repetitive electrical stimulation model established by stimulating the dura mater in conscious rats can simulate the chronification of frequent onset of acute migraine, from the perspective of cutaneous allodynia and nociceptive behaviors. PACAP appears to have a part in the pathogenesis of migraine potentially via PAC1 receptor. PACAP is co-expressed with CGRP, and therefore shows the potential to be considered a new therapeutic target for migraine [102]. The use of electrical stimulations led to the development of some clinical trials in which their use for the treatment of migraine has been assessed [103,104].

Furthermore, the effect of electroacupuncture (EA) pretreatment has been investigated. Pei et al. conducted a study regarding the use of EA in rats [105]. In this study, the authors used a conscious rat model of migraine induced by repeated electrical stimulation of the dura mater. Animals treated with EA showed an increase in exploratory, locomotor and eating/drinking behavior and a reduction in freezing-like resting and grooming behavior. In animals that received dural stimulation, an increase of c-Fos neurons in the periaqueductal grey, raphe magnus nucleus, and TNC was observed. This study showed that EA pretreatment may reduce behavioral responses to electrical stimulation of the dura mater in a rat model of recurrent migraine. These findings suggest that EA pretreatment may improve migraine-like symptoms by altering the descending pain modulatory system. Notwithstanding, further molecular and electrophysiological research is needed to better comprehend the central mechanisms of EA treatment of migraine [105].

Further emerging therapeutic targets are the acid-sensing ion channels (ASICs), which are considered neuronal proton sensors. Amiloride (a non-specific ASIC blocker) is a compound that showed to have benefic effects in animal models of migraine. Verkest et al., investigated the involvement of the ASIC1-subtype in cutaneous allodynia [106]. The authors conducted an investigation on effects of systemic administrations of amiloride and mambalgin-1 (a specific inhibitor of ASIC1a- and ASIC1b-containing channels) on cephalic and extra-cephalic mechanical sensitivity. The treatment was performed on a rat model of acute and CM induced by intraperitoneally administration of isosorbide dinitrate. The systemic administration of these compounds reversed cephalic and extra-cephalic acute cutaneous mechanical allodynia, whereas a single administration caused a delay in the subsequent establishment of chronic allodynia. Established chronic allodynia was also reversed by both mambalgin-1 and amiloride. A single daily administration of mambalgin-1 also showed to have a preventive effect on allodynia chronification. Pharmacological results obtained in this study suggest the involvement of peripheral ASIC1-containing channels in cutaneous allodynia and in its chronification. Furthermore, the results indicate the therapeutic potential of ASIC1 inhibitors in acute and prophylactic migraine treatment [106].

The involvement of TWIK-related spinal cord K^+^ (TRESK) channels has also been investigated. These channels are expressed in TG and DRG neurons and are the major background K^+^ channels in primary afferent neurons. Mutations in TRESK channels have been associated with familial and sporadic migraine. Nevertheless, whether enhanced TRESK channel activity would reduce the excitability of primary afferent neurons has not been evaluated. Guo et al. [107] observed that the over-expression of TRESK subunits lead to an increase in background K^+^ currents, a reduction of input resistance, and a reduction in the excitability of small-diameter TG neurons. The overexpression of TRESK subunits inhibits capsaicin-evoked spikes in TG neurons, suggesting that a TRESK-specific channel opener may exhibit analgesic effect via reducing the excitability of primary afferent neurons [107].

Lengyel et al., investigated in in vitro the effects of chemically modified analogs of cloxyquin, tested on TRESK and other K_2P_ channels. Cloxyquin is known as a specific activator of TRESK (K_2P_18.1, TWIK-related spinal cord K^+^ channel) background potassium channel. In this recent study, among the modified analogues of cloxyquin used, the authors identified A2764, a selective inhibitor of TRESK that can inhibit TRESK in native cells, leading to cell depolarization and increased excitability. This compound may be of use to probe the role of TRESK channel in migraine and nociception [108].

## 5. Conclusions

Migraine is a disorder with a multifactorial etiopathogenesis in which the complicated pharmacological management requires further efforts to develop more efficacious therapies. Research advances strongly contributed in expanding our understanding of the pathways involved in its complex pathophysiology. Animal models have been developed on the base of clinical observations and some of them represent valuable predictive tools for the identification of anti-migraine drugs. Among the animal models developed to date, chemical provocations models based on the use of NTG or inflammatory soup have been the most widely used models to induce hyperalgesia and inflammation. The opioid receptor agonists such as SNC80, ARM390 or JNJ20788560, are revealing to be effective in counteracting hyperalgesia and CSD, respectively induced by chemical provocation using NTG and KCl. Nanoparticulate drug delivery systems might represent novel avenues to improve drug efficacy in brain targeting. In these novel formulations, triptans are encapsulated in nanoparticulates in order to better exert their pharmacological activity in brain by crossing the BBB. Furthermore, new emerging classes of medications, including 5-HT receptor agonists (ditans), CGRP receptor antagonists (gepants) and receptor or ligand antagonists (mAbs) are opening further options in therapeutics for EM and CM. Despite the medications already used in migraine therapeutics, further efforts are required to improve research in the translational pharmacological approach, from animal models to humans, in order to develop new therapeutic strategies.

## Figures and Tables

**Table 1:** Current pharmacological treatment in migraine.

Medication	Mechanism of Action	Potential Common Side Effects	Ref.
NSAIDs (nonsteroidal anti-inflammatory drugs)	Inhibition of the synthesis of prostanoids; cyclooxygenase enzymes (COX-1 and/or COX-2) inhibitors.	Nausea or vomiting, dyspepsia and diarrhea.	[13,14]
Ergotamine and dihydroergotamine (DHE)	Agonist to: 5-HT -1B, -1D and -1F receptors; D -1, -2, dopamine receptors. Partial agonists to alpha -1A, -1B, 1D adrenergic receptors.	Nausea and vomiting.	[13,14]
beta-blockers (metoprolol, propranolol, timolol)	beta -1, -2, -3, adrenergic receptors blockers.	Bradycardia, hypotension, nausea, diarrhea, bronchospasm, dyspnea, fatigue, insomnia, and dizziness.	[13,14]
Triptans	Agonists to 5-HT -1B,1D receptors	Dizziness, fatigue, dry mouth, flushing, feeling hot or cold, chest pain.	[13,14]
Opioids	Agonist µ opioid receptors	Constipation, itchiness, and nausea.	[13,14]
Antiepileptic drugs (divalproex sodium, valproate sodium, topiramate)	Sodium channel blockade Calcium channel blockade GABA agonism/potentiation	Nausea, vomiting, dizziness, diarrhea, drowsiness, constipation and dry mouth.	[13,14]
Antiemetics (metoclopramide, prochlorperazine)	Dopamine receptor antagonists (at D1, D2, D3 and D4 receptors)	Fatigue, constipation, ringing in the ears, dry mouth, restlessness, and muscle spasms.	[13,14]

**Table 2:** New emerging treatments in migraine.

New Drugs	FDA Evaluation	Administration	Mechanism of Action	Dosage	Response to Treatment	Common AEs	Ref.
Erenumab	Approved	SC	IgG2 CGRP receptor blocker	70 or 140 mg monthly	The percentage of patients with at least a 50% reduction in migraine days per month, was about 40% and 50% for both dosages in EM and CM.	Injection site pain, URI, fatigue, Nasopharyngitis, constipation, nausea.	[43,44]
Fremanezumab			IgG2 CGRP ligand antagonist	225 mg monthly or 675 mg every 3 months	For both dosages the percentage of patients with at least a 50% reduction in migraine days per month, was about 40% of CM and 45 % for EM.	Injection site erythema, injection site induration, diarrhea, anxiety, depression.	[45]
Galcanezumab			IgG4 CGRP ligand antagonist	240 mg loading dose (2 subsequent injections of 120 mg)	The percentage of patients with at least a 50% reduction in migraine days per month was about 30% of CM and 60 % for EM for both dosages.	Nasopharyngitis, URI, diarrhea, injection site pruritus, injection site erythema.	[46]
Eptinezumab	Not yet approved	IV	IgG1 CGRP ligand antagonist	Dosage ranges 30 to 300 mg	For EM, decreased the average number of migraine-days (30 mg = −4.0; 100 mg = −3.9; and 300 mg = −4.3). For CM, the mean change in migraine days was −8.2 in the 300-mg group.	Nausea, influenza, dizziness, fatigue, URI, UTI.	[47]
Rimegepant		Oral	CGRP receptor agonist	Dosage ranges 10 to 600 mg	At 2 h post dose, freedom from the most bothersome associated symptoms (MBS) and freedom from pain were reached. Improvements in functional disability were observed, with many patients reporting normal function.	Nausea, UTI.	[48,49]
Ubrogepant			CGRP receptor agonist	Dosage ranges 50 to 100 mg	For both doses, the percentage of patients free from pain at 2 h post administration was about 20% and the percentage of patients free from MBS at 2 h post administration was about 40%. In addition, phonophobia and photophobia resolution at 2 h post administration was reached.	Nausea, somnolence, dizziness.	[50]
Lasmiditan			5-HT_1F_ receptor agonist	Dosage ranges 50 to 200 mg	The percentage of patients free from headache 2 h post dose was about 30% for 50 and 100 mg, and about 40% for 200 mg. The percentage of patients free from MBS 2 h post dose was about 40% and 45% for 50 and 100 mg, and about 50% for 200 mg. In addition, photophobia and phonophobia resolution was reached.	Dizziness, somnolence, paresthesia, fatigue, and nausea.	[51]

**Table 3:** Current animal models of migraine.

Animal Models	Route of Administration	Description	Response to Treatment	Ref.
Inflammatory soup	Dural cannulation	Mechanical hyperalgesia; reduced locomotor activities; nociceptive behavior; unilateral hind paw; facial grooming; anxiety- and depression-like behaviors; altered CGRP-related genes in the TG and TNC.	Zolmitriptan reduced nociceptive behaviors. Ketorolac reduced the nociceptive behavior, ipsilateral hind paw and facial grooming. Amitriptyline reversed the allodynia and decreased depression- and anxiety-like behaviors.	[56,57,83,84]
NTG	Intravenously or intraperitoneally	Mechanical hyperalgesia; thermal and mechanical allodynia; photophobia; meningeal blood flow; reduced locomotor activities; facial expressions of pain.	Propranolol, topiramate, and amiloride inhibited mechanical hyperalgesia. Valproic acid were ineffective. Sumatriptan inhibited hypoactivity and grimace scale scores in rats, but resulted in hyperalgesia.	[64,85,86,87]
MOH	Intraperitoneally	Mechanical hyperalgesia; CSD-related bioelectrical alterations; activation of inflammatory markers in the TG.	Topiramate blocked the enhanced Fos expression in the TNC and inhibited cutaneous allodynia.	[66,88,89]
Genetic model	-	Spontaneous episodic trigeminal allodynia.	Valproic acid prevented the spontaneous changes in trigeminal allodynia.	[78]
Transgenic models (FHM-1, FHM-2)	-	Photophobia; unilateral head grooming; lateralized winking/blinking.	Rizatriptan reduced head grooming.	[73,77]
CSD model	Injections of KCl on the cerebral cortex, or electrical or mechanical stimulation of the cortex	Induced dural activation, vasodilation of meningeal blood vessels. Enhanced neuronal activation at the level of trigemino nuclear complex and in higher cerebral areas of the trigeminal pain pathway.	Valproate, topiramate, propranolol, amitriptyline and methysergide were shown to suppress SD susceptibility. Lamotrigine was also shown to block KCl-induced CSD.	[90,91]

**Table 4:** New compounds and nanoparticles in experimental models.

Experimental Models	Compounds	Type of Administration	Response to Treatment	Ref.
CM (NTG-induced)	SNC80	IP	The administration of the compound showed in all four models to induce a reduction of peripheral and cephalic allodynia	[92]
PTH (closed head weight drop method combined to NTG)				
MOH (sumatriptan-induced)				
OIH (morphine-induced)				
NTG model	SNC80 or ARM390 or JNJ20788560	IP	Reduction of NTG-evoked hyperalgesia	[93]
	Gas-D	IG	Reduced head-scratching behavior	[100]
CSD (KCl-evoked)	SNC80	IP	Reduced number of CSD events	[93]
Electrically evoked meningeal PPE	NOX-L41	IV or SC	Inhibition of neurogenic meningeal PPE	[94]
Behavioral studies	RB-SLNs	Orally	The brain uptake potential was 18.43-folds higher with respect to the pure drug in its free form, 2 h post the drug administration. Higher effectiveness in minimizing the number of writhings than the standard pure drug group. Enhanced time spent by animals in the light compartment of the light/dark box model (*p* < 0.001).	[95]
	SS-chitosan SLNs		The brain uptake potential was 4.54-folds increase in drug targeted to brain, compared to plasma, after 2 h of drug administration. A reduction of the number of writhings (*p* < 0.001) and enhanced time spent in lit box of light/dark box model (*p* < 0.001) compared to control groups was observed.	[96]
	SS-BSA-ApoE NPs		The brain uptake potential of SS was 12.67-folds higher compared to controls, 2 h post drug administration. Reduced writhings events compared to control groups. Enhanced tolerance to light in the light compartment of the light/dark box model compared to controls.	[97]
	ZNPs		An increase of 14.13-folds of drug that reached the brain compared to the pure drug was observed. The treatment reduced significantly the number of writhings compared to control (*p* < 0.001). Significant reduction (*p* < 0.001) of photophobia was achieved by enhancing the time spent in lit compartment of the light/dark box model.	[98]
	Nystatin-NPs	IP	Major accumulation of NPs in the brain than the other organs considered i.e., liver and spleen, indicating that nanoformulation was successful in reaching the brain through i.p. administration. The nanoformulation induced a decrease in the number of writhings in the acetic acid induced writhings test compared to controls (*p* < 0.001). The time spent in lit compartment by animals treated with Nystatin-NPs was higher than controls (*p* < 0.001), indicating the successful brain targeting through its nanoformulation.	[99]
Model of nociceptive durovascular trigeminal activation	Gastrodin, ligustrazine	IV	Gastrodin showed to inhibit nociceptive dural-evoked neuronal firing in the TCC. Ligustrazine showed no relevant effect on spontaneous activity in the TCC.	[101]

## Abbreviations

AEs	Adverse Effects
SC	Subcutaneous
IV	Intravenous
IP	Intraperitoneal
IG	Intragastrically
URI	Upper Respiratory Infection
UTI	Urinary Tract Infection
EM	Episodic Migraine
CM	Chronic Migraine
TGVS	Trigeminovascular System
TNC	Trigeminal nucleus caudalis
CGRP	Calcitonin gene-related peptide
DHE	Dihydroergotamine
5-HT	Serotonin
CNS	Central nervous system
DRG	Dorsal root ganglia
TG	Trigeminal ganglion
TCC	Trigeminocervical complex
CLR	Calcitonin receptor-like receptor
RAMP1	Receptor activity-modifying protein 1
RCP	Receptor component protein
mAbs	Monoclonal antibodies
PGE	Prostaglandin
BBB	Blood-brain-barrier
NTG	Nitroglycerin
MOH	Medication overuse headache
FHM	Familial hemiplegic migraine
KI	Knock-in
CSD	Cortical spreading depression
DOR	Delta opioid receptor
PTH	Post-traumatic headache
OIH	Opioid-induced hyperalgesia
PPE	Plasma protein extravasation
RB-SLNs	Rizatriptan benzoate-loaded solid lipid nanoparticles
ZNPs	Zolmitriptan nanoparticles
SS	Sumatriptan succinate
SLN	Solid lipid nanoparticles
NPs	Nanoparticles
PBCA	Poly (butyl cyanoacrylate)
BSA-ApoE	Bovine serum albumin linked with apolipoprotein E3
Gas	Gastrodin
